# Thrombospondin-1 is a multifaceted player in tumor progression

**DOI:** 10.18632/oncotarget.19165

**Published:** 2017-07-11

**Authors:** Tingting Huang, Li Sun, Xianglin Yuan, Hong Qiu

**Affiliations:** ^1^ Department of Oncology, Tongji Hospital, Tongji Medical College, Huazhong University of Science and Technology, Wuhan, Hubei, PR China

**Keywords:** thrombospondin-1, tumor progression, tumor angiogenesis, cancer cell behavior, tumor immunity

## Abstract

Thrombospondins are a family of extracellular matrix (ECM) proteins. Thrombospondin-1 (TSP1) was the first member to be identified and is a main player in tumor microenvironment. The diverse functions of TSP1 depend on the interactions between its structural domains and multiple cell surface molecules. TSP1 acts as an angiogenesis inhibitor by stimulating endothelial cell apoptosis, inhibiting endothelial cell migration and proliferation, and regulating vascular endothelial growth factor bioavailability and activity. In addition to angiogenesis modulation, TSP1 also affects tumor cell adhesion, invasion, migration, proliferation, apoptosis and tumor immunity. This review discusses the multifaceted and sometimes opposite effects of TSP1 on tumor progression depending on the molecular and cellular composition of the microenvironment. Clinical implications of TSP1-related compounds are also discussed.

## INTRODUCTION

Extracellular matrix (ECM) is a complex three-dimensional network of secreted proteins that is constantly remodeled due to the balance between synthesis, deposition and degradation [1]. The complex interplay between cells and ECM provides an environment that fosters cancer progression. Thrombospondins (TSPs) are a family of ECM proteins widely present in embryonic and adult tissues, which consist of five members: TSP1, TSP2, TSP3, TSP4, and TSP5 [2].

TSP1 was the first member to be identified, and is a main player in tumor microenvironment. Its role as an angiogenesis inhibitor was confirmed by numerous potential drugs against angiogenesis-driven diseases. Loss of TSP1 expression was regarded as an “angiogenic switch” from the non-angiogenic phenotype in tumor progression [3]. In addition to angiogenesis modulation, the effects of TSP1 on tumor progression are multifaceted and sometimes opposite depending on the molecular and cellular composition of the microenvironment [4]. Indeed, TSP1 regulates diverse processes such as adhesion, invasion, migration, proliferation, apoptosis, immunity response and treatment response by interacting with multiple ligands [4]. Consequently, strategies blindly targeting a specific function may result in severe adverse effects and the beneficial properties of the multifunctional TSP1 may also be lost. Here, we will focus on TSP1 and its function in tumor progression. Clinical implications and further directions for targeting TSP1 are also discussed.

### The structure and function of TSP1

### Structural and functional domains of TSP1

**Figure 1:**
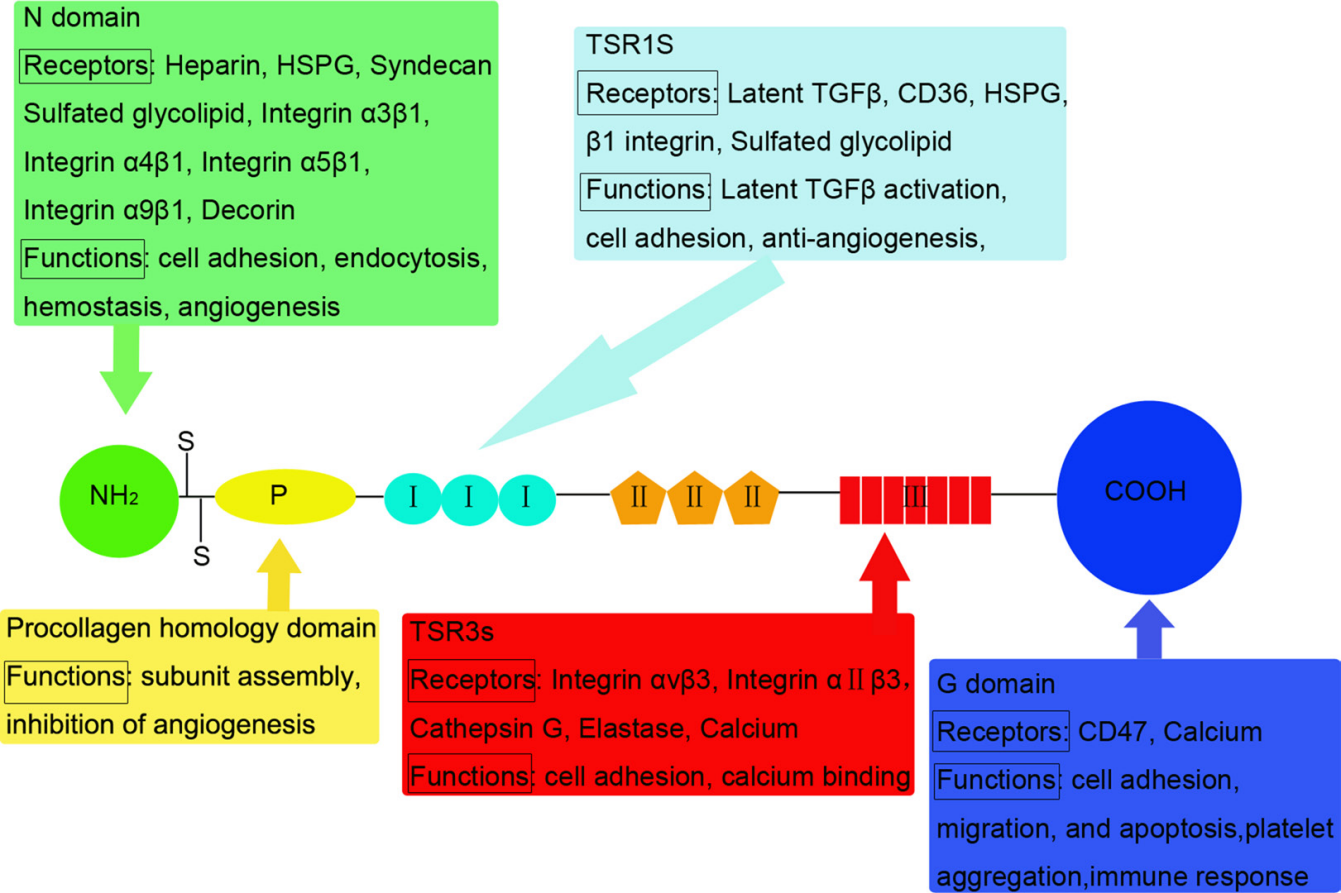
Structural and functional domains of TSP1: Mature polypeptide chains are linked by disulfide bonds TSP1 monomer comprises of N and G domains and a thin connecting strand. Interactions between structural domains and multiple cell surface molecules determine the diverse functions of TSP1. Receptors and functions that have been identified for the various domains are listed below or above the motif.

TSP1 is a 450-kDa homotrimeric glycoprotein. Mature polypeptide chains contain 1152 amino acids (180 kDa), linked by disulfide bonds between cysteine 252 and 256 [5]. Each TSP1 subunit comprises of N- and C-terminal globular domains (N and G domains) and a thin connecting strand (Figure 1) [6, 7]. The N domains arise from the N-terminal of the three polypeptidic segments. Each segment contains a heparin-binding domain (HBD) [8, 9]. The affinity between TSP1 and heparin is used to purify TSP1 from platelets [10]. Adjacent to the N-terminal, there is a region that is homologous to procollagen. This domain is involved in the assembly of the protein into a trimer [2]. The role of this domain remains unknown but could be used to block the angiogenic process [11]. A motif repeated three times constitutes type I repeats (TSR1s) framed around six cysteinyl residues. Different active sequences in the TSR1s play different roles in tumor progression [12]. Type II repeats (TSR2s) constitute three motifs of 60 amino acids containing six cysteinyl residues as in TSR1. The precise role of the domains has not been reported in cell surface receptors interactions or cell growth regulation [2]. The seven type III repeats (TSR3s) are rich in aspartic residues and responsible for calcium binding. They interact with αvβ3 and αIIβ3 integrins in various cell types [13]. The G domain, also called cell-binding domain (CBD), is specific to the TSP family (TSP1-5) [14]. A peptide from the G domain has been proposed to stimulate platelet aggregation [15]. The cell binding peptide from the CBD is integrin-associated protein (IAP or CD47) [16].

### Interactions of TSP1with its receptors

The diverse functions of TSP1 depend on the interactions between its structural domains and multiple cell surface molecules (Figure 1). The receptors for TSP1 include: low density lipoprotein receptor-related protein (LRP), proteoglycans and sulfatides, CD36, integrins, integrin-associated protein (IAP), and unidentified receptors for G domains [17]. Most domains could bind multiple receptors, indicating crosstalk between the receptor systems.

### TSP1 in tumor angiogenesis

Angiogenesis, an important event in many diseases including cancer, is the process by which new blood vessels are formed from pre-existing vasculature [18]. The new blood vessel formation during tumor angiogenesis enhances tumor growth and provides an opportunity for cancer cells to metastasize [19]. Regulation of angiogenesis depends upon the balance between proangiogenic and antiangiogenic factors. There is controversy as to the exact biological function of TSP1 in angiogenesis. Although some studies suggest that TSP1 promotes neovascularisation [20], TSP1 is commonly recognised as an endogenous angiogenesis inhibitor.

### Domain-specific angiogenic activity

The specific domains of TSP1 exert angiogenic activity. In the study by Tolsma et al. [11], a series of protease-generated fragments were tested by several assays that reflect angiogenic activity. They found that the majority of the antiangiogenic activity of TSP1 resides in the procollagen homology domain and TSR1s. Short peptides from these two domains could block the angiogenic process both *in vivo* and *in vitro*. Dawson et al. substituted one of three L-amino acids by their D-enantiomers in Mal II (a 19-residue peptide derived from TSR1s that was inactive in angiogenesis assay) [21]. Substituted peptides inhibited the migration of capillary endothelial cells, and showed potent antiangiogenic activity. DI-TSP, a modified version of Mal II, markedly suppressed the growth of endothelial cells *in vitro* and reduced microvessel density *in vivo* [22]. Another study showed that the second and third TSR1s but not the procollagen homology domain inhibited angiogenesis by chorioallantoic membrane angiogenesis and endothelial cell proliferation assays [23]. The expression of 4N1K peptide derived from the G domain significantly correlates with reduced tumor angiogenesis [24, 25]. Furthermore, the heparin binding 25kDa fragment of TSP1 is responsible for the angiogenic activity. Conversely, the 140kDa fragment lacks angiogenic activity, and is a potent inhibitor of FGF2-induced angiogenesis [26]. Ferrari and colleagues reported that TSP18 (a recombinant 18kDa protein from the N domains of TSP1) accelerated tube formation of human umbilical vein endothelial cells (HUVECs) [27]. So the role of TSP1 in angiogenesis may rely on the tumor environment. The effect on angiogenesis will differ depending on which part of TSP1 is functional in a given setting.

### Stimulation of endothelial cell apoptosis

TSP1 modulates the apoptosis of endothelial cells that are forming new vessels. Guo *et al*. demonstrated that peptides from TSR1s elicit endothelial apoptosis [28]. The mechanism for inducing apoptosis by TSP1 peptides did not require ceramide generation, which mediated apoptosis in many cell types due to various stimuli. The peptide-induced apoptosis was mediated by dephosphorylation, since tyrosine and serine-threonine phosphatase inhibitors significantly blocked bovine aortic endothelial cell apoptosis [28].

TSP1 binds numerous receptors, and CD36-TSP1 interaction is considered to be important as a negative regulator of angiogenesis [29]. HUVECs transfected with a CD36 expression vector had a slower growth rate than parental cells [30]. TSP1-induced apoptosis was sensitive to antibodies that neutralized TSP1 or blocked access of TSP1 to CD36. Treatment of endothelial cells with an antibody that activates CD36 induced apoptosis, verifying the role of CD36 in this response. Melanoma cells seeded in the lungs of nude mice seldom developed into tumors large enough to see with the naked eyes if the mice were systemically treated with TSP1. There was a considerable increase in the percentage of apoptotic endothelial cells in the TSP1-treated group as compared to the saline-treated group, mainly in the area of active neovascularization surrounding the tumor [31]. The CD36-dependent signaling pathways through which TSP1 mediates apoptosis was elucidated by the activation of CD36-p59^fyn^-caspase 3-p38MAPK cascade, c-Jun N-terminal kinases and Fas/Fas ligand [31–33].

TSP1 might also induce the apoptosis of endothelial cells by the lack of hemodynamic forces or irregular flow conditions [34, 35]. Hemodynamic forces were essential for the survival of vascular endothelial cells. The disturbance of laminar flow also induced apoptosis. These investigations showed that the apoptosis was induced by the autocrine loop of TSP1 and the αvβ3 integrin/IAP complex in both cases. However, evidence demonstrated the administration of αvβ3 antagonists led to the regression of angiogenic blood vessels [36]. This appeared to be inconsistent with the concept that TSP1, a ligand of αvβ3, could induce apoptosis. At present, TSP1-directed receptor(s) which promotes endothelial cell survival during angiogenesis remains unclear.

### Inhibition of endothelial cell migration and proliferation

Endothelial cell migration and proliferation are important for the formation of sprouting capillaries. CD36 is necessary for the inhibition of endothelial cell migration and tube formation by TSP1. Antibodies against CD36 prevented TSP1 from inhibiting migration. Large vessel endothelial cells that lacked CD36 became sensitive to TSP1-mediated inhibition of migration after CD36 transfection [30]. Cyclic TSP1 mimetics had higher potency in human microvascular endothelial cells (HMVECs) compared to HUVECs, which was likely due to the difference in expression of CD36 in HMVECs as compared to large vessel cells such as HUVECs [37]. While other researchers found TSR inhibited vascular endothelial growth factor (VEGF)-induced HUVEC migration via a CD36-independent pathway. TSR was unable to inhibit VEGF-induced migration using a β1 integrins-activating antibody or by suppressing β1 integrins, indicating that β1 integrins was necessary for TSP1-mediated inhibition of migration. They raised that β1 integrins could regulate cell migration via a PI3K-dependent, Akt-independent pathway [38]. These data are consistent with a prior observation that the TSP1 TSRs were pan-specific ligands for β1 integrins [39]. Both CD36 and β1 integrins may differentially contribute to TSP1-mediated migration inhibition depending on the microenvironment and the migration stimulus.

TSP1 induces growth arrest in endothelial cells by suppressing the cell cycle [40, 41]. Yamauchi *et al.* suggested that TSP1 induces cell-cycle arrest through upregulation of p21 expression mediated by p53 [40]. The proliferation of HMVECs could be inhibited by the interaction of TSP1 with the very low density lipoprotein receptor (VLDLR). This process was not mediated by CD36 and TSRs [41]. Other mechanisms by which TSP1 inhibits endothelial cell proliferation need thorough exploration.

### Regulation of VEGF bioavailability and activity

VEGF is a multifunctional cytokine that contributes to angiogenesis by both direct and indirect mechanisms. VEGF is overexpressed in a high percentage of malignant animal and human tumors [42]. The expression levels of VEGF and TSP1 are used to describe angiogenesis in different tumor samples. Upregulation of TSP1, together with downregulation of VEGF in cancer cells, might play a role in the hypovascularity of cholangiocarcinoma compared to hepatocellular carcinoma [43]. Increased VEGF-A and decreased TSP1 in carcinomas as compared to adenomas were associated with the malignant phenotype [44]. Microvessel count showed a significant positive correlation with the expression of VEGF and an inverse correlation with TSP1 in papillary thyroid carcinoma [45]. VEGF increased proliferation and migration of pituitary endothelial cells, while TSP1 suppressed these effects [46]. Breast tumors in a TSP1-rich environment could markedly increase the secretion of VEGF that counterbalance the inhibitory effect of TSP1 [47]. These findings indicate that the levels of VEGF and TSP1 are indicators of angiogenesis but do not explain if one regulates the expression of the other.

Mutation of the tumor suppressor gene p53 has been associated with the increase of VEGF expression and the decrease of TSP1 expression [48–50]. However, no association was found between p53 mutations and TSP1 in non-small cell lung carcinoma. While, a significant association was found between p53 mutations and high VEGF expression and neovascularization [51]. More patients are needed to prove an association between p53, VEGF and TSP1 expression in cancer.

### Impact of TSP1 on cancer cell behaviors

### Adhesion

Cell adhesion to ECM is a crucial step in tumor progression and metastasis. In 1987, TSP1 was first shown to function as a cell adhesive protein [52]. Thereafter, many studies have demonstrated that TSP1 mediates cellular adhesion of numerous cell types, regardless of species.

Integrins are a family of cell surface glycoproteins that play a major role in cell adhesion. The α3β1 integrin, with the cooperation of sulfated glycoconjugates and α4β1, was the domain integrin mediating adhesion of breast cancer cells to TSP1 [53]. Other studies showed that TSP1 favors direct MDA-MB-231 adhesion via αvβ3 and α6 integrins [54, 55]. The αvβ3 integrin also mediated melanoma cell adhesion to TSP1 [56]. TSP1 was an adhesive protein for the human small cell lung carcinoma (SCLC) cell lines. The two classic SCLC cell lines, OH-1 and H128 attached only on substrates coated with TSP1. SCLC cells adhesion to TSP1 was mediated by interactions of TSP1 with both α3β1 integrin and sulfated glycolipids [57]. TSP1 could promote cell substrate adhesion to osteosarcoma cells through the α4β1 integrin. The adhesion to TSP1 was inhibited by antibodies against the α4 or β1 subunit but not by antibodies against other integrins [58]. CD36 was the first nonintegrin receptor for TSP1 to be described. TSP1 overexpression up-regulated CD36, leading to enhanced adhesion of human cutaneous squamous cell carcinoma cells to TSP1 [59].

However, TSP1 has variable effects in tumor cell adhesion. TSP1 decreased tumor cell adhesion through upregulation of urokinase plasminogen activator receptor (uPAR)-controlled urokinase plasminogen activator (uPA) and plasmin activities [60]. These data suggest that TSP1 may have a dual role in tumor cell adhesion. The intrinsic proadhesive capacity of TSP1 plays a significant role in maintaining the cell-cell and cell-matrix interactions needed for local growth of tumors. When TSP1 overrides its intrinsic proadhesive effect, it promotes detachment of tumor cells from primary tumor, invasion of surrounding tissues, and metastasis.

### Invasion and migration

Metastasis is a multistep event that allows tumor cells to invade through ECM and migrate to distant organs. The precise role of TSP1 in tumor invasion and migration remains controversial, with compelling evidence suggesting both stimulatory and inhibitory roles.

In the study by Pal et al., TSP1 enhanced the invasion and migration of oral squamous cell carcinoma cells and stimulated the expression of MMPs partly via the integrin signaling, which cooperatively facilitated cancer invasion [61]. The positive feedback between TSP1 and TGFβ provided a versatile system to rapidly build the tumor microenvironment [61–63]. Increased expression of TSP1 was associated with the invasive and metastatic phenotypes of melanoma and invasive ductal carcinoma of breast [64–66]. TSP1 was a potent stimulator of prostate tumor cell migration, and this effect required CD36, which also mediated TSP1 antiangiogenic activity [67]. Both oncogene and tumor- suppressor gene activation in cancer are associated with the regulation of TSP1 expression. PTEN or BRAF was involved in TSP1-induced invasion and migration of thyroid cancer cells [68, 69]. TSP1 regression by oncogenic Myc might be a critical determinant of metastatic phenotypes in medulloblastoma [70]. In our previous study, FGFR2-induced upregulated TSP1 was associated with the invasion and migration of human gastric cancer cells [71] .

An *in vivo* study of transgenic mice with breast cancer showed that tumors in TSP1-null mice grew faster than in wild-type mice. At 90 days of age, the number of metastatic lesions in lung was higher in the wild-type than TSP1-null mice [72]. TSP1 inhibited migration of clear cell renal carcinoma cells in response to different stimuli [73]. Esophageal squamous cell carcinoma patients with low expression of TSP1 were associated with worse progression-free survival. Furthermore, TSP1 inhibited the migration signaling in both cancer cells and surrounding endothelial cells *in vitro* and *in vivo* [74]. These results reinforce the pleiotropic nature of TSP1, which depends on the tumor microenvironment, and the presence of its different receptors may have different, even opposite, effects on cell behavior and biological process.

### Proliferation and apoptosis

The first *in vivo* analysis of the impact of TSP1 in an intestinal carcinogenesis model at early stages of tumor initiation and development showed a decrease of TSP1 expression in adenomas, decreased tumor cell apoptosis, and its inverse relationship with more proliferative and vascularized intestine [75]. Similarly, azoxymethane/dextran sodium sulfate induced tumors developed in TSP1^-/-^ colons had higher proliferation index. TSP1 interacted with proteins implicated in proliferation, DNA repair, and transcriptional regulation such as protein arginine methyltransferase 6 [76]. Interestingly, proliferation only occurred when breast cancer cells were exposed to lower concentrations of TSP1, and increasing the TSP1 concentration attenuated cell proliferation [77]. In human prostate cancer and cutaneous squamous cell carcinoma, TSP1 did not directly affect proliferation and apoptosis of cancer cells [59, 78]. TSP1 expression was an independent prognostic factor for clear cell renal cell carcinoma, and was strongly correlated with proliferation index [79]. In contrast, Miyata *et al.* were unable to show any significant correlation between TSP1 and various clinicopathological features or tumor size. The 4N1K peptide from TSP1 exhibited significant association with microvessel density, apoptotic index and tumor size [24]. Differences in the populations, histological subtypes, and antibody used in these studies may explain the discrepancies.

### TSP1-mediated modulation of tumor immunity

The ability of cancer cells to evade or escape the immune response is recognized as cancer hallmark, which provides a platform for treatments based on immunotherapies [80]. The first suggestion that TSP1 may influence the immune response of cancers came from the observation that TSP1 and its receptors played a role in monocyte-mediated killing of squamous carcinoma [81]. Several lines of evidence also suggested that TSP1 promoted M1 macrophage recruitment and cytotoxicity [82, 83]. Thus, avoiding this innate immune surveillance could provide a selective pressure to reduce TSP1 expression during tumor progression. Importantly, TSP1-primed monocytes upregulated TGFβ levels and successfully inhibited inflammation in the intestine. Since inflammatory bowel disease (IBD) is characterized by chronic inflammation in the intestinal tract and carcinogenesis is promoted by persistent chronic inflammation occurring in IBD, understanding the underlying mechanisms is essential in order to ameliorate inflammation and prevent colorectal cancer [76, 84]. However, not all macrophages or monocytes residing in tumors are tumoricidal. Further work is needed to determine whether TSP1 can enhance or suppress tumoricidal activities of these cells.

The effect of TSP1 on early natural killer (NK) cells proliferation was related to activation of TGFβ because anti-TGFβ neutralizing antibody completely abrogated TSP1-mediated inhibition of early NK cell proliferation. However, active TGFβ added only at culture initiation increased late NK cell expansion similar to TSP1 [85]. These data suggest that besides TSP1/TGFβ, additional factors are important for the regulation of NK cell proliferation.

TSP1 has been reported to induce regulatory T (Treg) cells. In human melanoma, anti-TSP1 mAb strongly inhibited CD4^+^ Treg induced by snail. Survival was significantly prolonged by the anti-TSP1 mAb in mice. When TGFβ mAb was injected, no antitumor effect was observed. Since TSP1 is known to be a TGFβ activator, these phenomenon suggested that TSP1 would be a more potent effector molecule than TGFβ in the snail-induced immunosuppression [86].

The binding of TSP1 to CD47 controlled adaptive immunity via inhibiting T-cell activation and differentiation [87]. TSP1 ligation to CD47 further controlled immune responses by regulating maturation and trafficking of dendritic cells (DCs) [88–90]. Cutaneous administration of TSP1 shRNA or adoptive transfer of TSP1-deficient DCs produced anti-tumor effects by modulating the immune response in animal tumor models [91]. These TSP1/CD47 pathways have pathophysiological consequences of immune challenges including in anti-tumor immunity.

Based on recent progress, the known and unknown mechanisms of TSP1 in tumor immunity are the major problems that limit the improvement of anti-tumor immune responses in cancer patients.

### Clinical implications

Therapeutic strategies targeting TSP1 have been extensively reviewed [4, 92]. Generally, three approaches are used to increase TSP1-mediated inhibition of tumor progression: (1) TSP1-derived peptides, recombinant fragments and mimetics; (2) antibody blockade and gene therapies; (3) upregulation of the potential effects of endogenous TSP1. Numerous peptides and modified structural agents derived from TSP1 have been developed. Here, we only describe some preclinical and clinical drugs, especially those that have been tested in clinical trials (Table 1).

**Table 1:** TSP1-related compounds that have been tested in clinical trials

Compound	Origin	Stage	References
ABT-510	TSR1s	Phase II	92–96
CVX-045	TSP1 mimetic+antibody	Phase I	103–104
Trabectedin	Marine natural product	Approved	105–108

### ABT-510

ABT-510 is the only agent derived from TSP1, which was conducted in phase II clinical trials. It is a 9-amino acid synthetic peptide that has a single D-amino acid replacement in one properdin region heptapeptide. Phase I clinical studies suggested that ABT-510 was safe and well tolerated [93, 94]. A small phase II study of ABT-510 for the treatment of metastatic melanoma did not demonstrate definite clinical efficacy. However, ABT-510 caused significant decrease of peripheral blood VEGF-A and VEGF-C levels. Limited increase in the frequency of peptide specific cytotoxic T cells was also observed [95]. Phase II study in patients with previously untreated advanced renal cell carcinoma showed little evidence of clinical activity for ABT-510 and further evaluation of ABT-510 as a single agent in renal cell carcinoma was unwarranted [96]. In another phase II study in patients with advanced soft tissue sarcoma, ABT-510 had a favorable safety and tolerability profile.

The rates of disease control and overall survival were encouraging. But the low objective response rate and lack of dose response limited these results as compelling evidence of strong single-agent activity [97]. These responses in clinical trials failed to provide clear evidence of efficacy when used as monotherapy. Importantly, the half life of ABT510 is only one hour in human plasma. ABT-510 is no longer in clinical development.

### ABT-898

ABT-898, a second generation of TSP1 mimetic peptide, has enhanced stability, increased half life of 4-5 hours and decreased side effects or toxicities [92]. In a mouse model of human late stage epithelial ovarian cancer and experimental prolactinomas in female rats, ABT-898 induced tumor regression and decreased tumor vasculature [98, 99]. ABT-898 had more notable antitumor activity compared to ABT-510 in dogs with soft tissue sarcoma [100].

ABT-898 is currently being tested as monotherapy against canine sarcoma. Unfortunately, ABT-898 with sufficiently slow clearance has not been tested in human diseases. Results indicated that ABT-898 was potent in the female reproductive tract, including promoting follicular atresia, inducing ovarian cancer regression, and inhibiting endometriosis in mouse models [98, 101, 102]. In addition, its anti-inflammatory effect in a colitis model may be a new therapeutic alternative in inflammatory diseases [103]. Thus, more clinical trials focused on female reproductive disease and inflammatory diseases are needed in filling in unmet medical needs.

### CVX-045

CVX-045 is a monoclonal antibody fused to TSP1 mimetic peptides, which not only preserves anti-angiogenic property but also extends half life. The preclinical data suggested that CVX-045 exhibited significant anti-angiogenic activity in several tumor models and enhanced anti-tumor ability in combination with chemotherapy or targeted therapy [104]. No immunogenicity and dose-limiting toxicities were encountered in phase I results for 18 patients with advanced solid tumors. However, two patients experienced severe adverse events attributed to CVX-045. Only one patient demonstrated partial response and 33% patients showed stable disease for at least 8 weeks. Limited benefits along with severe adverse events prevent its farther development [105].

### Trabectedin

Trabectedin (Yondelis, ET-743), a marine natural product, has been approved for the treatment of advanced or metastatic soft tissue sarcoma and relapsed ovarian cancer. The underlying mechanism remains unclear. Dossi *et al*. demonstrated that trabectedin showed antiangiogenic activity linked to the upregulation of TSP1, which might represent a potential marker for response to trabectedin [106]. Trabectedin is currently undergoing phase II clinical trials for several other tumors. Trabectedin was well tolerated but lacked activity in pretreated advanced-stage colorectal cancer and metastatic pancreatic adenocarcinoma [107, 108]. Single-agent trabectedin had moderate activity in HER2-overexpressing metastatic breast cancer [109]. More clinical trials of trabectedin combined with chemotherapy, radiotherapy or targeted-therapy are needed in the future.

### Conclusion and future directions

**Figure 2:**
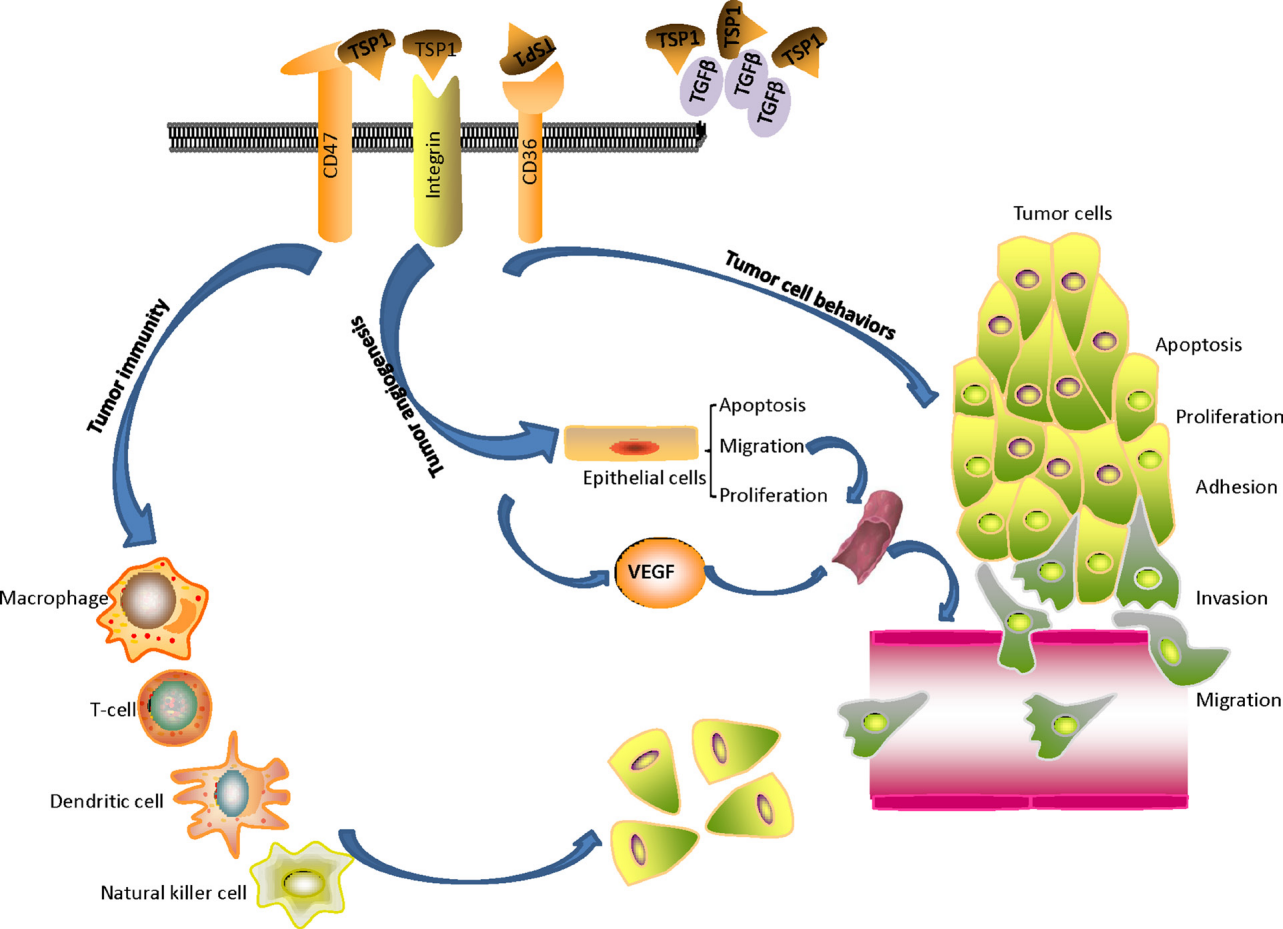
The role of TSP1 on tumor progression: TSP1 binds to multiple membrane proteins, causes direct assembly of complexes, and affects signal transduction that regulates tumor progression TSP1 acts as an angiogenesis inhibitor by stimulating endothelial cell apoptosis, inhibiting endothelial cell migration and proliferation, and regulating vascular endothelial growth factor bioavailability and activity. TSP1 affects tumor immune response, tumor cell behaviors including adhesion, invasion, migration, apoptosis, and proliferation.

The role of TSP1 in tumor progression is summarized in Figure 2 and Table 2. TSP1 binds to multiple membrane proteins, causes direct assembly of complexes, and affects signal transduction that regulates cell proliferation, invasion, migration, apoptosis, differentiation, etc. The correct identification of the TSP1 interaction networks will give us a better understanding of its role and mechanisms of how it acts with interacting molecules to affect biological and pathological processes in cancer.

**Table 2:** Impact of TSP1 on cancer cell behaviors

Author	Cancer	TSP1 expression	Impact of TSP1 on cancer cell behaviors	Prognosis
Chandrasekaran *et al.*^53^ Gomes *et al.*^54^ John *et al.*^55^	Breast cancer	-	Pro-adhesive activity	-
Albo *et al.*^60^	Breast cancer	-	Promoted invasion Decreased adhesion	-
Ndishabandi *et al.*^72^	Breast cancer	-	Promoted tumor growth and decreasedmetastasis	-
Hyder *et al.*^77^	Breast cancer	-	Promoted proliferation (low concentration) Decreased proliferation (high concentration)	-
Horiguchi *et al.*^65^	Invasive ductal carcinoma of the breast	Increased (IHC)	Promoted lymph node metastasis	-
Sipes *et al.*^56^	Melanoma	-	Pro-adhesive activity	-
Borsotti *et al.*^64^	Melanoma	Increased (microarray analysis)	Promoted invasion and metastasis	-
Jayachandran *et al.*^66^	Melanoma	-	Promoted invasion, chemoresistance andmesenchymal phenotypes	-
Guo *et al.*^57^	SCLC	-	Pro-adhesive activity	-
Decker *et al.*^58^	Osteosarcoma	-	Pro-adhesive activity	-
Streit *et al.*^59^	Cutaneous squamous cell carcinoma	-	Pro-adhesive activity No effect on proliferation and apoptosis	-
Pal *et al.*^61^	Oral squamous cell carcinoma	Increased (IHC and cDNA microarray )	Promoted migration and invasion	-
Motegi *et al.*^63^	Oral tumor	-	Promoted migration	-
Seliger *et al.*^62^	Glioma	-	Promoted migration	-
Firlej *et al.*^67^	Prostate cancer	-	Promoted migration and invasion	-
Goel *et al.*^78^	Prostate cancer	-	No effect on proliferation and apoptosis	-
Nucera *et al.*^68^	Papillary thyroid carcino-ma	-	Promoted invasion and metastasis	-
Soula-Rothhut *et al.*^69^	Thyroid cancer	-	Promoted migration and invasion	-
Zhou *et al.*^70^	Medulloblasto-ma	-	Promoted migration and invasion	-
Huang *et al.*^71^	Gastric cancer	Increased (IHC)	Promoted migration and invasion	-
Bienes-Martinez *et al.*^73^	Clear cell renal Carcinoma	-	Decreased migration and invasion	-
Zubac *et al.*^79^	Clear cell renal Carcinoma	Decreased (IHC)	Decreased proliferation	Survival
Tzeng *et al.*^74^	Esophageal squamous cell carcinoma	Decreased (IHC)	Decreased migration and metastasis	Progression-free survival
Gutierrez *et al.*^75^	Intestinal adenoma	-	Decreased proliferation and promoted apoptosis	-
Lopez-Dee *et al.*^76^	Colorectal cancer	-	Decreased proliferation	-

IHC: The expression of TSP1 was examined by immunohistochemical analysis in cancer tissues.

Therapeutic interventions that either upregulate or downregulate TSP1 may prove useful in the future. Researchers should focus on small molecules that allow a more accurate regulation, thus leading to limited adverse effects and optimal responses. Translation of these small molecules into the clinic and identification of optimal combined strategies with chemotherapy and radiotherapy will be further challenges. Development of innovative therapeutic strategies targeting TSP1 will likely continue for many years and promises to be a fascinating voyage of discovery.
